# Beyond the Binary: Sexual and Reproductive Health Considerations for Transgender and Gender Expansive Adolescents

**DOI:** 10.3389/frph.2021.670919

**Published:** 2021-10-06

**Authors:** Claire E. Lunde, Rebecca Spigel, Catherine M. Gordon, Christine B. Sieberg

**Affiliations:** ^1^Biobehavioral Pediatric Pain Lab, Department of Psychiatry and Behavioral Sciences, Boston Children's Hospital, Boston, MA, United States; ^2^Pain & Affective Neuroscience Center, Department of Anesthesiology, Critical Care, and Pain Medicine, Boston Children's Hospital, Boston, MA, United States; ^3^Nuffield Department of Women's and Reproductive Health, Medical Sciences Division, University of Oxford, Oxford, United Kingdom; ^4^Division of Adolescent/Young Adult Medicine, Boston Children's Hospital, Boston, MA, United States; ^5^Harvard Medical School, Boston, MA, United States; ^6^Department of Psychiatry, Harvard Medical School, Boston, MA, United States

**Keywords:** adolescent, sexual health, transgender, reproductive health, gender-affirming care, gender expansive

## Abstract

Awareness and visibility of transgender individuals have grown exponentially. However, conceptualizing sexual and reproductive health (SRH) as “women's” or “men's” health services further marginalizes transgender and gender-expansive (TGE) youth. Multiple reviews and commentaries have been published on the topic of SRH care for adults under the umbrella term of sexual and gender minorities, all with a call to action for more inclusive care and the need for more clinical research involving TGE individuals, and notably, TGE youth. Results from adult TGE studies are often translated to describe adolescent models. However, models specific to adolescent TGE populations are needed to understand their unique SRH needs. This review will describe the current literature relating to SRH needs of TGE youth and adults, highlighting key areas with significant disparities in need of further research. This comprehensive summary will also provide recommendations for clinicians and researchers with the goal of improving SRH care and obtaining wider representation in both clinical settings and research directed toward TGE youth.

## Case Study

In May 2019, the *New England Journal of Medicine* published a case study describing the power and limits of classifying sex and gender within medicine (1). Sam, 32 years old, presented to an emergency department with severe and intermittent abdominal pain. Despite disclosing his transgender identity, the electronic medical system indicated his affirmed gender, and therefore, medical staff evaluated him as a man with a non-urgent condition. Several hours later, Sam delivered a stillborn baby. This tragic case begs the following questions: What are the healthcare experiences of transgender and gender-expansive (TGE) individuals with sexual and reproductive health (SRH) needs? What is the empirical evidence supporting the needs of this population? What is the frequency of cases like Sam's? Most importantly, what do clinicians and researchers need to learn from this case to prevent SRH injustices from reoccurring? Selected studies were compared and summarized by the authors, leading to our suggestions and recommendations for treating TGE youth, which may then prevent adults, such as Sam, from experiencing poor medical care.

## Introduction

SRH healthcare is often conceptualized as “women's” or “men's” health services, which may be excluding many people from seeking care. Using the inclusive umbrella term will allow health care professionals to use an organ inventory as opposed to the currently used, often binary, options (2). Health care professionals should be sensitive and understand how gender, as opposed to sex assigned at birth, can directly affect clinical practice. Those who identify as transgender have a gender identity that differs from the sex that was assigned at birth; those who identify as non-binary may have a more complex, fluid, multifaceted, or otherwise less clearly defined gender than a transgender person (3). Those who identify as transgender can be further divided into two groups: transgender women (assigned male at birth) and transgender men (assigned female at birth). Although often incorporated into the same broad “sexual and gender minority” category, TGE individuals have healthcare needs that are distinct from those who identify as lesbian, gay, and bisexual (LGB). LGB issues are often *interpersonal*, related to their relationships with others, while gender issues are *intrapersonal*, with an emphasis on an individual's identity and self-expression. This distinction is rarely emphasized in the literature (4). Furthermore, TGE individuals have unique SRH health needs, but are often excluded from gynecological and reproductive practices, as current guidelines and recommendations exist within a gender binary, heteronormative system, catering care to those who identify as heterosexual and cisgender (5). Awareness and visibility of transgender individuals have exponentially increased over recent years. Multiple reviews and commentaries have been published on the topic of SRH care under the umbrella term of “sexual and gender minorities”, (6–16) and include a call to action for more inclusive care and clinical research involving TGE individuals. However, TGE youth face unique barriers in receiving care and this subset population remains vastly under-researched. This review will discuss barriers to care for TGE adolescents and young adults, emphasizing the unique SRH care needs of this population and ultimately providing recommendations for U.S. clinicians and researchers with the goal of improving SRH care for TGE youth.

The prevalence of TGE individuals, particularly adolescents, remains relatively undefined and existing data are likely underestimated (17, 18). The American College of Obstetrics and Gynecology Committee Opinion published in March 2021 estimated 150,000 youth and 1.4 million adults living in the U.S. identify as transgender (19). Previous estimates include data from: (1) The 2011 National Transgender Discrimination Survey (20) which included 6,450 participants ages 18 to 89 and found that 33% of respondents did not identify as exclusively male or female, and 14% identified as gender-nonconforming; and (2) The 2015 U.S. Transgender Survey (18) which included 27,715 participants over 18 years old with 31% identifying as non-binary. As social stigma decreases and education surrounding sex and gender increases, (21) the prevalence of adolescents identifying as a gender other than cisgender has been increasing (3% in 2018) (22). However, according to the Harris Poll, (23) 2019 societal acceptance decreased and was accompanied by an increase in LGBTQ discrimination because of sexual orientation or gender identity (24). This is thought to be a product of a lack of non-discrimination laws for the LGBTQ community and the political and cultural divide brought on by the election year of 2016. Transgender, and specifically non-binary adolescent populations, are underrepresented in clinical and biomedical research and as a result, vast knowledge gaps exist.

## The Problem: Barriers to Care

TGE youth encounter significant interpersonal and structural barriers to receipt of high-quality healthcare, including discrimination, lack of clinician knowledge and health systems obstacles. TGE youth often experience social stigma, harassment, and rejection, and unlike adults, TGE youth are embedded in families and schools which may make such stigma difficult to escape (25). Health care settings are often sites of mistreatment, where discriminatory practices range from refusal of care to verbal, physical or sexual abuse (26). A secondary analysis from the 2015 U.S. Transgender Survey (*N* = 19,157) found that almost one-quarter of transgender adults avoided healthcare due to anticipated discrimination, with the highest prevalence among transgender men (27). The intimate nature of SRH makes this issue particularly acute: 33% of transgender people and 48% of transgender men have delayed healthcare or avoided preventive measures (*e.g.*, pelvic exams or STI screening) out of fear of discrimination or disrespect (28). Compared to cisgender, LGB-identifying individuals, transgender adults are more likely to delay care and report negative effects of disclosure to their clinician (29). Racial disparities also exist, as transgender people of color experience significantly higher levels of transphobic discrimination compared to their White counterparts in accessing health services (30). Barriers to care are even more pronounced and complex for transgender youth who are reliant on parental support and insurance coverage for care. A 2016 study aimed at understanding perceived barriers to care among transgender youth (ages 14–22 years) and their caregivers identified six themes (31): (1) few accessible pediatric providers are trained in gender-affirming health care; (2) lack of consistently applied protocols; (3) inconsistent use of chosen name/pronoun; (4) uncoordinated care and gatekeeping; (5) limited/delayed access to pubertal blockers and cross-sex hormones; and (6) insurance exclusions.

The lack of adequate clinician training is one of the most often cited barriers to quality care for TGE populations (32). The 2011 National Transgender Discrimination Survey found that 50% of respondents reported having to teach their clinicians about their own healthcare needs that may be specific to TGE individuals, such as hormone usage, reproductive conditions and menstruation suppression (20). Researchers have responded by surveying clinicians about their knowledge and attitudes towards caring for TGE individuals. (33, 34) A 2015 survey of 141 obstetrician-gynecologists across nine academic hospitals found that 80% did not receive training in residency regarding the care of transgender patients; only 35% and 29% felt comfortable caring for male-to-female and female-to-male transgender patients, respectively (33). The findings from another recent study are equally discouraging (35); of the 169 board-certified obstetrician-gynecologists surveyed in 2018, less than half (43%) reported training specific to the healthcare needs of LGB or TGE individuals. Moreover, this study found previous clinician training was not associated with increased comfort in taking care of TGE patients. Mounting research suggests that increasing hours of TGE health education is not sufficient; rather, future training should address personal biases and internalized transphobia (36).

Structural inequities imbedded within the healthcare system can limit access to healthcare for transgender people. Many transgender individuals lack health insurance, which may be partially due to the higher prevalence of unemployment and poverty faced by these individuals relative to the general US population (37). TGE adolescents and young adults are at a further disadvantage, as they are reliant on their parents for not only insurance coverage (31, 38) but also support of their gender identity, which they often do not have (39). As a result, transgender individuals, particularly youth, are disproportionately represented in the homeless population (40). Even for those individuals and families who are insured, barriers persist, as private insurers have historically excluded coverage for medical interventions related to gender affirmation and transition (41). A study examining barriers to gender transition-related healthcare in Massachusetts found that mental health coverage emerged as one of the factors most strongly associated with an inability to access care (42). Previously, a diagnosis of gender dysphoria by a mental health professional was required before approval was granted to cover gender affirming therapy or surgery (43). With new informed consent models, patients are able to access gender-affirming hormones through their primary care clinician without a diagnosis of gender dysphoria, (44) which is the feeling of discomfort or distress that might occur in people whose gender identity differs from their sex assigned at birth or sex-related physical characteristics. However, not all clinics use informed consent models, and most insurers still require a diagnosis for those seeking gender affirming surgery. Similarly, electronic health records (EHRs) may pose as a structural barrier if gender identity is not included as an important variable to care. For example, birth sex auto-populates medical recommendations for clinicians that may be needed for a person assigned female or male at birth during specific ages (e.g., menstruation preparation, prostate exam). Including both sex and gender as important clinical variables in EHRs could aid in decreasing both structural and interpersonal barriers for TGE youth and young adults when receiving SRH care (45). Advocacy work is needed to eliminate roadblocks to accessing medically necessary transition-related care for TGE youth across the U.S (46).

These barriers to healthcare result in increased health risk and an increased likelihood of negative health outcomes for an already vulnerable population. The consequences of inadequate care are staggering. According to one retrospective cohort study, 56% of youth who identified as transgender reported previous suicidal ideation, and 31% reported a previous suicide attempt, compared with 20% and 11% among matched youth who identified as cisgender, respectively (12). The social and economic marginalization and abuse experienced by TGE individuals has been shown to increase these health risks (47). It is imperative that clinicians, researchers and public health officials collaborate to mitigate these barriers and improve both access and quality of care for this population.

## The Role of the Obstetrician-Gynecologist

### Current Expectations

As highlighted above, lack of adequate provider training often poses a significant barrier to TGE individuals obtaining sensitive SRH care. Thus, gynecologists striving to provide inclusive SRH care should be informed on the reproductive health needs and considerations of *all* genders, not only those who are cisgender. In 2011, the American College of Obstetricians and Gynecologists assembled a committee to assist gender-diverse individuals in obtaining routine treatment and screening, as well as gender-affirming care needs (48). Transgender males who have internal and external female anatomy will need standard SRH care such as contraception counseling, reproductive health education, breast, and gynecologic cancer screenings, STI testing, and menstrual management. However, it is important for obstetrician-gynecologists to understand the various options for hormonal and surgical gender affirmation, as these interventions impact the SRH needs and considerations of TGE individuals. The following section first defines medical options to affirm gender and thereafter explores implications across key SRH domains.

### Medical Options to Affirm Gender

Delaying pubertal changes through administration of gonadotropin-release hormone (GnRH) agonists, may decrease discomfort and improve quality of life while creating time and space for gender identity exploration (7). New evidence suggests that affirming interventions during adolescence improve psychosocial outcomes, such as decreasing suicidality and increasing attainment of higher education, employment, and reliable housing (49). Pubertal suppression may also reduce the need for later surgery because physical changes that are otherwise irreversible (protrusion of the Adam's apple, male pattern baldness, voice change, breast growth, etc.) are prevented (50). Gender affirming hormones (GAH), (i.e., testosterone and estrogen), enable TGE individuals to gain characteristics that match their gender identity. The most recent Endocrine Society guidelines indicate that medications can be started in patients younger than 16 years old (51). A wide range of gender-affirming surgeries (previously referred to as gender reassignment surgeries) are also available to TGE individuals such as feminizing vaginoplasty, masculinizing phalloplasty, metoidioplasty (clitoral enlargement), as well as face and voice procedures (52).

### Sexual Desire and Function

TGE youth who may or may not be receiving medications for transition are often exploring their sexual identity and sexual pleasure. TGE receiving GAH, such as pubertal suppression and cross-sex hormones, may experience side effects from these medications that impact their sexual health. For example, a multicenter one-year prospective clinical trial of 53 trans men and 53 trans women receiving GAH showed a significant increase in sexual desire and clitoral pain in trans men receiving testosterone (53). Trans women in this study reported decreased sexual desire as a side effect of decreased testosterone concentrations. TGE youth taking pubertal suppression medication may also experience decreased libido as a side effect (54). Gender affirming surgeries also impact sexual functioning. A study examining the outcomes of vaginoplasty, the procedure that restructures the head of the penis into a clitoris and creates a vaginal cavity, found that 90% of trans females who had the surgery reported that they were still able to have an orgasm with 75% reporting that their orgasms were either the same or more intense than before (55). Research on procedures for trans men (e.g., phalloplasty and metoidioplasty), have shown generally positive outcomes; however, for some decreased phallic sensation and the inability to penetrate negatively impacted sexual wellbeing (56). Those using puberty blockers or testosterone/estrogens may be navigating changes to libido. When counseling trans men, clinicians should discuss how patients might develop pleasurable sex and how erogenous stimulation may have to be rediscovered. Youth, particularly TGE youth, show variable amounts of comfort with discussing physical intimacy and sexual intercourse with peers, partners, and providers and may not even know what questions to ask (57).

### Menstruation

Menstruation may lead to dysphoria for TGE youth with female anatomy (58). A recent survey of TGE adults with masculine identities showed mixed attitudes toward menstruation, with most reporting feeling unsafe and uncomfortable using men's restrooms during times of menstruation (59). Medications that lead to cessation of menses can be an important first step in gender-affirming care and may be used on their own or with GAH. This may be helpful for TGE youth who do not have access to GAH therapy, as there are no documented adverse outcomes, and only benefits reported with respect to an individual's quality of life (60–63).

### Contraception

All transgender people who have gonads and engage in sexual activity that could result in pregnancy should be counseled on the need for contraception (64). GAH should not be used as a form of contraception. Particularly, testosterone, a known teratogen, may lead to abnormalities of physiological development of the embryo if a patient becomes pregnant (65). Unintended pregnancies have occurred in transgender men receiving testosterone. Therefore, contraceptive needs should be addressed, especially in patients who have maintained their uterus (66). Moreover, some TGE individuals may be at risk for engaging in high-risk sexual behavior. Specifically, TGE youth are at high risk for sexual abuse and engaging in commercial or survival sex, (67) increasing the need for appropriate contraceptive counseling and sexually transmitted infection protection. The interpersonal and structural barriers to high-quality healthcare outlined above have led to contraception access issues, including the inability to afford services, lack of clinicians with expertise in gender-affirming therapy, difficulty in securing health insurance, and misconceptions about unintended pregnancy (68). Health care professionals, and those who care for adolescents, need to be aware of who may require and should receive advice about pregnancy prevention and contraception. Rather than assuming a patient's sexual orientation, clinicians should take a thorough sexual history based on the patient's identity and anatomy, with the understanding that gender identity does not determine sexuality (69). Additionally, clinicians and researchers should be aware and sensitive to issues that may affect gender and sexual health, notably gender affirming-hormone therapies that may affect fertility and libido.

### Pregnancy

Numerous studies have documented increased pregnancy rates in adolescent sexual and gender minorities compared with their heterosexual counterparts, with an estimated increased risk of adolescent pregnancy rate between 2 and 10 times (56, 70–72). This finding may be explained by a broad spectrum of sexual health risks experienced by TGE individuals, including an earlier age of first sexual intercourse, exposure to sexual abuse, and a higher number of sexual partners (73). TGE youth face conflict with their gender identity and potentially their sexual orientation. It is likely that their experience is similar to cisgender LGB adolescents as it pertains to reproductive health considerations. As outlined, the principles of obstetrical practice regarding pregnancy in a transgender male are not complex once a clinician has received appropriate training. However, the barriers to care and societal discrimination may cause pregnancy-related psychological distress. Two studies highlight both psychological issues experienced by transgender men contemplating pregnancy or bearing a child, as well as the unique medical implications for both the parent and fetus (66, 74). Similar to pregnancy research in TGE youth, few data exist on abortions in this population. Abortion-providing clinicians and advocates have long recognized the need for high-quality, gender-affirming care for their patients, although little is known about these services at facilities that provide abortions (75). Current abortion care should be adapted to include and affirm the experiences of this underserved population (76).

### Fertility Options and Attitudes

TGE individuals, similar to cisgender people, may want to become pregnant and reproductive desire has found to be high among transgender individuals. A recent Australian study surveyed 409 transgender and non-binary adults and found that, of participants who were not already parents, 33% hoped to have children in the future (77). A 2016 survey (*n* = 156) showed 71% of transgender and non-binary people surveyed were interested in adoption and 35.9% in biological parenthood (63). More gender nonconforming youth (44%) than transgender youth (26%) expressed interest in biological fertility. Medical interventions, such as GAH and surgeries, pose significant risk to reproductive potential and fertility outcomes. Specifically, the long-term effects of GAH on reproductive function are unknown (78). The World Professional Association of Transgender Health, the American Society for Reproductive Medicine, and the Endocrine Society recommend that all transgender patients be counseled on options for fertility preservation prior to initiating gender-affirming surgeries or hormonal transition (78–80). There are relatively limited data specific to fertility preservation for transgender individuals and current approaches are extrapolated from options for fertility preservation after oncologic diagnoses (80).

Options for individuals assigned female at birth vary by pubertal status and include embryo and oocyte cryopreservation, both of which are established in practice (81). Notably, both oocyte and embryo cryopreservation require controlled ovarian stimulation with repeated assessment by transvaginal ultrasound, which may be uncomfortable or emotionally distressing for these patients (80). Transgender men who have already initiated GAH therapy (testosterone) may still pursue oocyte or embryo cryopreservation (80). Recent findings have shown ovarian tissue cryopreservation to be a promising option. In 2019, the American Society of Reproductive Medicine opinion report on fertility preservation stated: “ovarian tissue cryopreservation is no longer considered experimental should be considered an established medical procedure” (82). A recent study showed that ovarian tissue does not seem to be morphologically affected by prolonged testosterone treatment; (83) however, there are conflicting data in the literature (84) and more research is needed in this area. Despite potential interventions, the use of reproductive options is surprisingly low (85). Some transgender adolescents have expressed discomfort with the idea of using their own body parts for reproduction (63) and oocyte cryopreservation has been shown to be associated with worsening gender dysphoria (86). Youth in these studies also identified cost, desire not to delay medical transition, and future plans to adopt children or no desire to have children as reasons for declining fertility preservation.

### Endometriosis

The SRH community has become increasingly aware of how TGE youth are experiencing complications of gynecologic organs. For example, transgender male adolescents have a uterus and ovaries leaving them susceptible to developing painful gynecological medical conditions such as endometriosis. This common gynecological disease, leads to chronic pelvic pain, decreased quality of life, (87) and has the proclivity to adversely affect choices surrounding fertility options. In addition, endometriosis negatively impacts social/romantic relationships and sexual intimacy (88). Endometriosis in adolescence, the typical period of symptom onset, (87) is an even more challenging problem as the disease may present with several clinical and pathological differences that are not observed in adult women (89). This disease affects at least 10% of cisgender women of reproductive age, although endometriosis also affects TGE people. To date, only one study has examined the prevalence, presentation, and impact of endometriosis in non-binary youth (90) with the conclusion that evaluation for endometriosis is underutilized when transgender youth present with pelvic pain and dysmenorrhea. Even for those with a known diagnosis, GAH therapy may not remedy endometriosis-associated pain. The impact of endometriosis must be considered for TGE youth in the context of the interpersonal and structural barriers to accessing SRH care, as outlined above. Further research is warranted regarding endometriosis and other gynecologic conditions in TGE individuals, particularly adolescents, to improve quality of care for this population.

## Recommendations for Providing Gender-Affirming SRH Care to TGE Adolescents

Adolescent medicine clinicians and researchers can lead the way in providing affirming and empowering care at a time when individuals are becoming aware of and developing their gender identities and sexual orientations. The gender-affirmative care model supports gender diversity as a normal part of human development, compared the others that may treat gender diversity as a disorder (6). The model uses strengths-based terminology, which emphasize an individual's self-determination and autonomy (Table 1). All clinical office staff also play a role in affirming a patient's gender identity. Frontline staff such as receptionists and clinical assistants can be involved in creating an affirming environment. Clinicians and staff should make direct and non-judgmental inquiries about a patient's experience and feelings before applying any labels. Thereafter, a patient-asserted name and pronouns should be used by staff and are ideally reflected in the electronic medical record without creating duplicate charts. In the clinical environment, making flyers available or displaying posters related to LGBTQ health issues, including information for children who identify as TGE and families, reveals inclusivity and awareness (50).

**Table 1:** Relevant terms and definitions defined by the *World Health Organization's glossary for terms and tools* for providing gender-affirmative care.

**Term**	**Definition**
Cisgender	Individuals whose gender identity corresponds with the sex the person had or was identified as having at birth
Gender Expression	The outward manner in which an individual express or displays their gender (i.e., clothing, hairstyle, speech, mannerisms)
Gender Expansive	A person's identity or behavior is broader than the commonly held definitions of gender and gender expression in one or more aspects of their life.
Gender Identity	A person's internal sense of self and how they fit into the world, from the perspective of gender
Gender Non-conforming	The state of one's physical appearance or behaviors not aligning with societal expectations of their gender (a feminine boy, a masculine girl, etc.).
Gender Non-binary	Individuals who do not identify their gender as man or woman. Other terms to describe this identity include genderqueer, agender, bigender, gender creative, etc.
Gender-fluid	A person has a flexible gender identity and expression that can fluctuate by the hour, day, or longer interval of time.
Sexual and Gender Minority	An umbrella term referring to individuals who identify as gay, lesbian, or bisexual, or who are attracted to or have sexual contact with people of the same gender and/or whose gender identity (man, women, other) or expression (masculine, feminine, other) is different from their sex (male, female) assigned at birth.
Sex	An individual's biological status as male, female, or something else. Sex is assigned at birth and associated with physical attributes, such as anatomy and chromosomes.
Sexual Orientation	Composed of attraction, behavior, and orientation. Refers to a person's sexual and emotional attraction to another person and the behavior and/or social affiliation that may result from this attraction (lesbian, gay, bisexual, etc.)
Transgender	Individuals whose current gender identity differs from the sex they were assigned at birth.

*The current definitions are listed in Table 1; however, these concepts may change as research continues. Additionally, it is important to consider these concepts as fluid as opposed to fixed categories*.

Support from a multidisciplinary and collaborative health care team would greatly improve health outcomes for TGE youth (91). Since 2007, when the first multidisciplinary gender clinic was established within a pediatric institution, (92) the number of such pediatric subspecialty programs has grown significantly (93). Despite the growing number of clinics, these valuable, affirming spaces remain inaccessible to some. Moreover, existing clinics often have high patient volumes leading to long waiting periods to be seen by a clinician. Gender affirming care may not always lead to gynecology referral as most care falls under the umbrella of primary care (69, 94). A 2016 study aimed at understanding perceived barriers to care among transgender youth (ages 14–22 years) and their caregivers also identified the following recommendations: (1) development of protocols for the care of young transgender patients, as well as roadmaps for families; (2) increased number of multidisciplinary gender clinics; (3) providing cross-sex hormones at an age that permits peer-congruent development; and (4) designating a navigator for transgender patients in clinics (31). An assessment of patient/caregiver satisfaction at a pediatric multidisciplinary gender clinic in Seattle, WA found that families were highly satisfied with multidisciplinary, coordinated health care with respondents endorsing the presence of a Care Navigator as integral to providing support to families during a process often fraught with barriers and emotional stress (95).

Re-conceptualizing outdated terminology may also serve to create an affirming environment. For example, endometriosis researchers have suggested relabeling the condition with the term “gendered disability”, as this is a more accurate depiction of the science and lived experiences of those with endometriosis (96). Additionally, researchers call for shifting the language used to describe endometriosis (96–98): (1) Discuss uteruses/ovaries/penises and other body parts without assigning them a gender; (2) Use phrases like “internal/external reproductive organs”; (3) Use language such as “people who menstruate/are pregnant/ produce sperm;” and (4) Use “cisgender” rather than “biologically female/male”; (5) Use people with endometriosis instead of women or patients; (6) Refer to endometriosis family/siblings/friends, instead of ladies/sisters/cysters; and (7) Discuss endometriosis as a system-wide, gendered disability that impacts the organs, nervous system, cognitive abilities, mood, respiration, circulation, and digestive functions as well as the reproductive system.

Sexual and gender minority people, including members of the lesbian, gay, bisexual, transgender, and queer communities, are understudied and underrepresented in research. Recent studies have shed light on the LGBTQ community's perspective on how SGMs are addressed in current studies (99). Findings from focus groups and qualitative studies have revealed: (1) sexual and gender questions did not allow for identity fluidity and complexity, reducing inclusion and representation; and (2) question stems and answer choices were often not clear as to whether gender identity or sexual orientation were being assessed (100). Measurement error from imprecise survey questions impose biases. A recent study aimed at the development of an affirming and customizable electronic survey of SRH experiences found that: (1) TGE community input from initial conceptualization to final implantation is essential; (2) investigators need to be mindful of gender-diversity and differences in sexual orientation when defining study eligibility criteria to directly reduce selection bias; and (3) the ability to use individualized, affirming, customized language for sexual and reproductive body parts and processes may avoid gender dysphoria evoked for some by medical terms (101). Lessons learned from these findings may inspire other researchers to innovate and think more inclusively, thereby creating affirming research for marginalized groups, such as TGE youth and adults. See Table 2 for a synthesized summary of published recommendations for clinicians and researchers with the benefit of the patient described.

**Table 2:** Suggestions for clinicians and researchers for providing care using the gender-affirming care model with the described patient benefit and source of recommendation.

**Recommendation**	**Benefit to patient**	**First Author (publication date)**
Use strengths-based terminology, which emphasize an individual's self-determination and autonomy boxes on patient intake forms and all medical records. Researchers should use inclusive language when recruiting study participants.	Reduce stigmatizing environment that hinders health care delivery.	1. Liszewski et al. (91) 2. Moseson et al. (75) 3.Tavits et al. (93) 4. Haseen et al. (9) 5. MacCarthy et al. (17)
Educate all healthcare staff, including clinicians, receptionists, etc. on becoming more gender-literate by receiving training in respecting patients' gender identity and gender expression	Establish open dialogue with patients, non-binary patients should not be obligated to educate health care professionals about their health care needs.	1. Liszewski (2018) (91) 2. Hodax et al. (7) 3. de Vries et al. (48) 4. Obedin-Maliver et al. (10) 5. White Hughto et al. (12)
Institutional nondiscrimination policies can be expanded to include gender identity and expression.	Processes for assessing patient satisfaction and lodging grievances should allow for confidential reporting of experiences relevant to patients' gender identity and expression.	1. Liszewski et al. (91) 2. Wang et al.(95) 3. Du Bois et al. (96)
The healthcare team should verbally express support, provide information on local support groups, educate teachers and school officials, and connect families to support organizations.	Decrease discrimination in school and lack support from teachers and peers which will then improve the patient's quality of life and potentially decrease depression and suicide in TGE adolescents. This may also decrease distress and associated psychological conditions and comorbid decrease often found in TGE adults.	1. Liszewski (2018) (91) 2. Wagner et al. (6) 3. Safer et al. (31)
The physical environment should also be optimized for inclusivity, such as providing gender-neutral restrooms, gender-neutral signage, and inclusive magazines.	Create safe spaces in waiting and examination rooms and communicate to the adolescent TGE patient that the healthcare team is an ally.	1. Liszewski et al. (91) 2. Moseson et al. (75) 3. Jones et al. (86)
Clinicians should use an “organ inventory” to document the presence or absence of specific organs to ensure appropriate screenings and evaluation	Assumptions about sex, gender, or anatomy can lead to inadequate care or a negative medical experience.	1. Liszewski et al. (91) 2. Deutsch et al. (97)
When taking a history, conducting an exam, or providing counseling, providers are encouraged to avoid using gendered terms.	Asking a patient what language they use to refer to their genitalia and menstruation, and mirroring that language, can help the provider avoid use of words that may be triggering.	1. Mehringer et al. (98) 2. Jones et al. (86) 3. Sczesny et al. (99) 4. Dinour et al. (100) 5. Likis et al. (101)
Adopt and advocate for electronic health records using a gender identity variable as well as birth sex. Gender identity should auto populate medical recommendations for clinicians that may be needed for a person identifying as TGE during particular ages.	Including both sex and gender as important clinical variables in EHRs could provide preventative care and aid in decreasing both structural and interpersonal barriers for TGE youths and young adults when receiving SRH care	1. Burgess (2019) (43) 2. Deutsch et al. (97) 3. Grasso et al. (102)

*Research and healthcare teams should maintain openness when introducing the gender-affirming model as many people do not fit or remain in a single category. As patients may change preferences, healthcare providers must continuously assess their current sex and gender inclusivity, language, and policies*.

## Discussion

Women's health and men's health should be described as sexual and reproductive health. TGE individuals have been largely excluded from clinical care and clinical research leading to large gaps in knowledge regarding their experiences, options, and needs in both settings. Extrapolating data from other populations (*e.g.*, solely transgender data, solely adult data) may not lead to accurate conclusions. Thus, research catering to TGE adolescents, young adults, and non-binary individuals, would be beneficial to the field of SRH. Obstetrician-gynecologists and other clinicians who specialize in gynecological care should assume their role in the care of TGE adolescents and young adults while maintaining awareness of the consequences of inadequate care, and the many existing interpersonal and structural barriers to receiving high quality SRH care. Adolescent clinicians and researchers can lead the way in providing affirming and empowering care at a time when individuals are becoming aware of and developing their gender identities and sexual orientations.

Inclusive clinical care and research that addresses the relationship between sex assigned at birth, gender identity and social determinants of health are crucial to achieving health equity (102). Commonly used or validated measures of sexual and reproductive health experiences typically use heteronormative and cis-normative assumptions and language, such as the types of sex people are having; (4, 103) the gender, sex assigned at birth, and current organs of people's partners; and their capacity for pregnancy (98). Clinical care and research are interconnected, as questions that arise in clinical care motivate and drive research, and research subsequently informs the standard of care and new healthcare policies. TGE youth greatly need wider representation in both settings.

## Author Contributions

CL and RS conducted the literature review and wrote the first draft of the manuscript. CG and CS contributed to manuscript preparation. All authors made a significant contribution to the work reported, whether that is in the conception, and took part in drafting, revising or critically reviewing the article gave final approval of the version to be published have agreed on the journal to which the article has been submitted and agree to be accountable for all aspects of the work. All authors contributed to the article and approved the submitted version.

## Funding

Support was provided by awards to CG – MCHP T71MC00009 LEAH training grant (HRSA/HHS) and R01 from the *Eunice Kennedy Shriver* NICHD (R01 HD101421); and CS – K23 Career Development (GM123372) & Supplement (GM123372-04S1) Awards from NIH, a Peer Review Medical Research Program Investigator-Initiated Research Award from the United States Department of Defense (W81XWH19105060); and support from the Boston Center for Endometriosis/Marriott Family Foundation.

## Conflict of Interest

The authors declare that the research was conducted in the absence of any commercial or financial relationships that could be construed as a potential conflict of interest.

## Publisher's Note

All claims expressed in this article are solely those of the authors and do not necessarily represent those of their affiliated organizations, or those of the publisher, the editors and the reviewers. Any product that may be evaluated in this article, or claim that may be made by its manufacturer, is not guaranteed or endorsed by the publisher.
